# Unveiling the Genetic Structure of New Caledonian Dugongs Using a Multiscale Genetic Approach: Conservation Challenges for an Isolated Population

**DOI:** 10.1002/ece3.72168

**Published:** 2025-09-14

**Authors:** Paolo Verger, Claire Garrigue, Claire Daisy Bonneville, Solène Derville, Marc Oremus, Camille Sant, Cécile Fauvelot

**Affiliations:** ^1^ Laboratoire d'Océanographie de Villefranche, LOV Sorbonne Université, CNRS Villefranche‐sur‐Mer France; ^2^ UMR ENTROPIE (IRD‐Université de La Réunion‐CNRS‐Ifremer‐Université de Nouvelle‐Calédonie) Noumea New Caledonia; ^3^ Association Opération Cétacés Noumea New Caledonia; ^4^ World Wildlife Fund Noumea New Caledonia

**Keywords:** effective size, environmental barrier, marine mammals, wildlife management

## Abstract

Coastal marine megafauna faces increasing threats from habitat degradation, climate change, and human activities, making conservation efforts crucial for their survival. The New Caledonian dugong population was reclassified as Endangered on the IUCN Red List in 2021, following research on its abundance and genetic diversity. With fewer than 800 individuals estimated between 2008 and 2012, urgent conservation measures are needed to prevent further decline. Modern genetic tools provide critical insights into spatial genetic differentiation and gene flow across New Caledonia's extensive lagoon habitats. In this study, we analyzed 66 skin samples from live and stranded dugongs collected between 2003 and 2023, using a multiscale genetic approach. We examined mitochondrial DNA control region sequences at the Indo‐Pacific level, 13 microsatellite loci to compare New Caledonian and Australian populations, and 2499 single nucleotide polymorphisms (SNPs) to assess fine‐scale structure within New Caledonia. Our findings confirm that the New Caledonian dugong population has extremely low genetic diversity and is highly differentiated from its Australian counterpart. The effective population size (*N*_*e*_) was critically low, ranging between 95 and 160 individuals, depending on the analytical approach. Within New Caledonia, we identified two genetically distinct clusters along the west coast, north and south of Bourail, a division consistent with previous satellite tracking studies showing no movement across this natural boundary. These findings highlight the urgency of conservation action and suggest that the population's isolation and low genetic diversity may warrant an upgrade to Critically Endangered status.

## Graphical abstract

The small New Caledonian dugong population is geographically isolated, with extremely low mitochondrial DNA. Using mitochondrial DNA, microsatellites, and SNPs from 66 samples collected over 2 decades, we identified fine‐scale genetic structure and confirmed strong differentiation from Australian populations. In addition, the New Caledonian dugong population has a critically low effective population size. These findings underscore the urgent need for targeted conservation and may support reclassification to Critically Endangered.

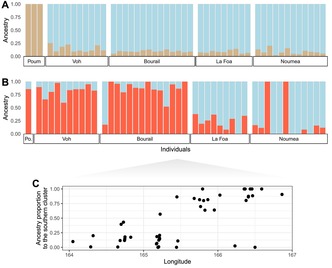

## 1 Introduction

A major concern in conservation biology is the impact of small population size on the genetics and demography of vulnerable species. While such species may have a broad distribution range and large populations overall, they often exist in small, isolated subpopulations, with some being at greater risk than others. Long‐term isolation and habitat fragmentation can severely affect the evolutionary potential of these small populations, reducing their evolvability—the ability of a lineage to adapt to novel environmental conditions (Kirschner and Gerhart 1998). This reduction in evolvability compromises the long‐term survival and sustainability of natural populations (Willi et al. 2006). Small, isolated populations are also more susceptible to inbreeding and genetic diversity loss, which can have severe repercussions on their fitness and viability. A striking example is the case of the three highly genetically isolated populations of the tammar wallaby ( *Macropus eugenii* ) on the Houtman Abrolhos Archipelago, Western Australia. These populations exhibit morphological abnormalities, which have been attributed to their low genetic diversity and high levels of inbreeding (Miller et al. 2011).

Sirenians are herbivorous marine mammals that play a crucial ecological role in marine and riverine ecosystems by influencing the biomass, productivity, and composition of macrophyte communities (Wirsing et al. 2022). Dugongs, *Dugong dugon* (Müller, 1776) Palmer, 1895, commonly known as “sea cows” are closely related to manatees. They are found in the tropical and subtropical waters of the Indo‐Pacific, from the coasts of East Africa to the western Pacific Ocean. Dugongs can live up to 70 years, with a generation time of approximately 20 years (Marsh and Sobtzick 2019). They inhabit coastal regions, typically in shallow waters, though predator avoidance strategies may lead them to seek refuge in deeper waters (Heithaus et al. 2002). Generally solitary, dugongs are sometimes seen in loose and unstable herds. Simple aggregations have been observed on feeding grounds without complex social interactions (Preen 1992). Large aggregations were also observed resting or basking at the surface of a habitat devoid of seagrass at low tide in winter in New Caledonia. This behavior is thought to be influenced by thermoregulation (Cleguer et al. 2024). Their movements are typically limited, with most staying within 15 km of seagrass beds (Sheppard et al. 2006). While large‐scale migrations are rare in this relatively sedentary species, some individuals have been known to travel over 100 km (Sheppard et al. 2006). As dugongs require warm waters at least 1 meter deep, their movements are closely related to tidal changes and seasonal temperature fluctuations (Sheppard 2008; Derville et al. 2022; Cleguer et al. 2024).

Despite their broad distribution, dugong populations have experienced significant declines worldwide in recent decades; the species is already extinct in Japan (Kayanne et al. 2022), the Maldives, Mauritania, and Taiwan (Marsh and Sobtzick 2019) and is considered functionally extinct in China (Lin et al. 2022). Even in Australia, which harbors the largest remaining dugong populations, local declines are being observed (Cleguer et al. 2023). This decline is largely attributed to anthropogenic pressures such as coastal development, industrial pollution, fishing activities, direct hunting, and the degradation and loss of seagrass habitats. The survival and well‐being of dugongs are intimately tied to the availability and health of seagrass meadows, which serve as their primary food source (Marsh et al. 2002) and habitat for key life stages such as mating (Infantes et al. 2020). As a result of past population declines over the entire distribution range of the species, dugongs have been listed as vulnerable on the IUCN Red List since 1982 (Marsh and Sobtzick 2019). As dugongs' habitats continue to deteriorate, the species faces increasing threats, making it imperative to understand both the environmental and genetic factors that contribute to their vulnerability.

Genetic studies on dugong have highlighted the need to assess their genetic status and vulnerability to ensure effective dugong conservation. Across their fragmented distribution range, dugongs are divided into several genetically differentiated lineages based on mitochondrial DNA (mtDNA) analyses: Each lineage is structured and strongly restricted to a limited geographical zone, and further subdivided into multiple sublineages (Blair et al. 2014; Plön et al. 2019; Poommouang et al. 2021; Garrigue et al. 2022; Furness et al. 2024). These local populations exhibit high levels of genetic differentiation and diverse evolutionary histories. Notably, western populations—restricted to relatively small areas—show genetic diversity levels up to 10 times lower than those observed in eastern Indo‐Pacific populations from Indonesia and Australia (Furness et al. 2024). In addition, they have experienced a significant decline in female effective population size over the last millennium (Furness et al. 2024).

Geographical and ecological barriers are known to contribute to dugong population fragmentation. While deep open waters act as major barriers between regional populations, more localized factors, such as ecological and oceanographic parameters, further reduce connectivity. For example, in the Australian population, connectivity is hindered by the fragmented distribution of seagrass beds, coastal urbanization, strong marine currents, and tidal fluctuations (McGowan et al. 2023). In addition to fragmentation, many populations exhibit low genetic diversity and signs of inbreeding, particularly in declining populations affected by habitat degradation (Marsh and Sobtzick 2019; Poommouang et al. 2022). Other local populations, such as those in the Comoros, Madagascar (Plön et al. 2019), and New Caledonia (Oremus et al. 2011, 2015), face additional challenges due to peripheral isolation. These small, isolated populations have reduced genetic diversity (Garrigue et al. 2022), which exacerbates their vulnerability.

In New Caledonia, the dugong population has recently been classified as Endangered by the IUCN, due to its small size, geographic and genetic isolation, ongoing decline from threats like illegal hunting and bycatch, and a high risk of catastrophic events with no possibility of repopulation from neighboring populations (Hamel et al. 2022). Aerial surveys conducted at the local level have provided alarming estimates of population size, revealing a worrying decline from approximately 1588 individuals in 2003 (Garrigue et al. 2008; revised Hagihara et al. 2018) to between 426 and 792 individuals during the period 2008 to 2012 (Cleguer et al. 2017; revised Hagihara et al. 2018). This decline is likely due to a combination of cumulative threats that directly and indirectly impact dugong survival and fitness. Identified causes of death from necropsies of stranded individuals in New Caledonia include poaching, boat collisions, entanglement in fishing gear, predation, and other natural factors (Garrigue et al. 2024). The high proportion of dugong mortalities linked to human activities is a predictable consequence of the inadequate design of Marine Protected Areas, which fail to sufficiently cover the species' core habitat in New Caledonia (Cleguer et al. 2015). The challenge of managing dugongs in this region lies in protecting a highly mobile and rare species within an expansive lagoon where seagrass resources are patchily distributed. Given the fragmented nature of both the habitat and population, understanding the spatial structure and gene flow is crucial for defining management units at an appropriate scale.

In this context, the present study aimed to enhance our understanding of the genetic composition of the New Caledonian dugong population by employing a range of molecular markers. Building on previous genetic analyses of this population based on mtDNA sequence analysis (Garrigue et al. 2022), this study expands the sample size and examines population structure across multiple spatial scales using nuclear markers (microsatellites and SNPs). Specifically, microsatellites were used to assess genetic divergence between New Caledonian and Australian populations, while SNPs provided insights into effective population size, kinship relationships, and fine‐scale genetic structure. We hypothesize that the New Caledonian population harbors lower genetic diversity than Australian dugongs due to its smaller size, exhibits signs of inbreeding, and has a critically small effective population size. Additionally, we expect a general pattern of panmixia, reflecting the dugong's ability to traverse large distances within the New Caledonian lagoon. This study will provide critical insights into the genetic health of this small, isolated population, including its risks of inbreeding or susceptibility to disease. These findings will be instrumental in refining conservation strategies and enhancing management measures to support the population's long‐term survival.

## 2 Material and Methods

### 2.1 Sampling

**FIGURE 1:**
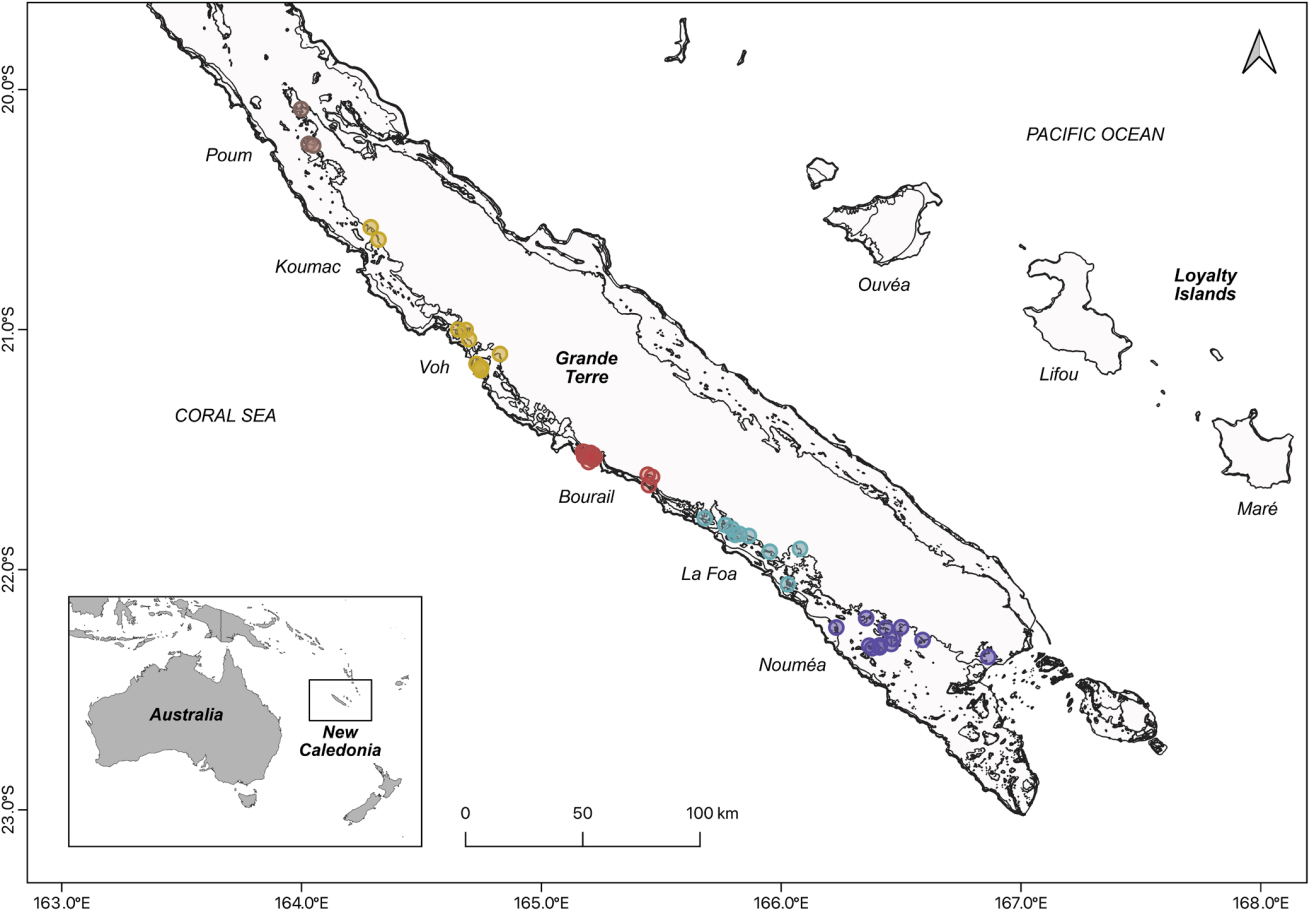
Geographical distribution of individual dugong samples along the coast of Grande Terre, New Caledonia. Sample points are color‐coded based on the nearest locality where they were collected, as indicated on the map. Land is shown in gray, and reefs are shown with a black contour line.

Between 2003 and 2023, 66 tissue samples were collected regularly from stranded dugongs (*N* = 37) or from animals that have been poached (*N* = 5) along the west coast of Grande Terre, the main island of New Caledonia (Figure 1, Table S1). In addition, biopsies were conducted on free‐ranging individuals during telemetry tagging operations in 2012, 2013, and 2019 (*N* = 24) along the west coast of Grande Terre (Table S1). All procedures involving live animals were conducted in accordance with relevant guidelines and regulations and approved by the competent animal ethics authorities. In total, 68 individual samples (Table 1) could be genetically analyzed. Samples were preserved in 75% ethanol at −20°C. The GPS coordinates were noted for each sampled individual (dead or tagged).

**TABLE 1:** Sampling details and genetic diversity indices for *Dugong dugon* in New Caledonia based on 13 microsatellite loci and 2499 SNPs.

*N*	Poum	Voh	Bourail	La Foa	Noumea	New Caledonia		
	4	11	17	15	19	66	North Queensland	South Queensland
MtDNA								
*n*	4	11	16	15	19	65		
*H*_*d*_	0	0	0	0	0.185 ± 0.110	0.061 ± 0.040	—	—
*π*	0	0	0	0	0.0001 ± 0.0002	0.0003 ± 0.0005	—	—
Microsatellite								
*n*	3	10	14	10	12	49	100	193
*A*_*R*_	1.26 ± 0.41	1.29 ± 0.33	1.33 ± 0.31	1.35 ± 0.29	1.33 ± 0.32	2.47 ± 1.28	7.11 ± 2.77	5.09 ± 1.91
*H*_*o*_	0.333	0.266	0.265	0.378	0.319	0.401	0.689	0.596
*H*_*e*_	0.111	0.292	0.329	0.352	0.333	0.427	0.725	0.601
*F*_IS_	−0.333	0.087	0.195*	−0.073	0.041	0.061	0.048	0.008
SNPs								
*n*	3	10	14	9	12	48	—	—
*H*_*o*_	0.369	0.341	0.324	0.340	0.337	0.337	—	—
*H*_*e*_	0.185	0.328	0.319	0.318	0.323	0.341	—	—
*F*_IS_	−0.990	0.016***	0.021***	−0.010	0.002*	0.023***	—	—

*Note:* *p* values of each test indicated as *p < 0.05; ***p < 0.001.

Abbreviations: *π*, nucleotidic diversity; *A*_*R*_, allelic richness (based on 49 microsatellite multilocus genotypes); *F*_IS_, fixation index; *H*_*d*_, haplotypic diversity; *He*, expected heterozygosity; *Ho*, observed heterozygosity; *n*, number of analyzed individuals; *N*, number of sampled individuals.

For data analyses requiring minimum sample sizes (i.e., population structure inference), samples were grouped based on their proximity to a known locality (Table 1): Noumea (including samples collected between Noumea and Prony, *N* = 19), La Foa (including samples from La Foa and Boulouparis, *N* = 15), Bourail (*N* = 17), Voh (including samples from Koumac and Voh, situated respectively to the north and south of the extensive seagrass bed on the Plateau des Massacres, *N* = 11) and Poum (*N* = 4).

### 2.2 Molecular Analysis

Approximately 25 mg of tissue was used to extract genomic DNA using a NucleoSpin Tissue kit (Macherey‐Nagel). Tissue was digested using 0.55 to 0.78 mg proteinase K and included an RNAse treatment. From the 66 available New Caledonian dugong samples, 55 have been previously analyzed in Garrigue et al. (2022) based on mtDNA sequences, and 33 have been previously analyzed by Oremus et al. (2015) based on 10 microsatellite loci. In this study, all 66 individuals were jointly reanalyzed in a common framework using a combination of various types of genetic markers: sex‐specific, mitochondrial, and nuclear markers.

The sex of the sampled individuals was determined using (1) the gametologs ZFY‐ZFX that were amplified with dugong‐specific primers (Dugong ZFX and Dugong ZFY; McHale et al. 2008) and (2) the male‐specific SRY gene was amplified with the dugong‐specific forward primer DSRY‐F and the elephant reverse primer ESRY‐R (McHale et al. 2008). PCR amplifications were conducted in a volume of 6 μL following the protocol described in Seddon et al. (2014) and run in a SimpliAmp Thermal Cycler device (Applied Biosystems, Foster City, USA) following their conditions. Amplification products were run on a 1.5% agarose gel, and sex was visually determined by comparing amplified and nonamplified profiles: amplification of the SRY gene produces a 153 bp fragment only in males, while amplification of the ZFY‐ZFX gametologs produces two 230 bp and 242 bp fragments in males and a single 230 bp fragment in females.

To infer broad‐scale phylogeographic patterns, we used a mtDNA marker, taking advantage of the extensive haplotypic data available in public repositories. Of the 66 individuals analyzed from New Caledonia, 53 had been previously genotyped by Garrigue et al. (2022). For the 13 additional samples collected between 2020 and 2023 (i.e., not included in Garrigue et al. 2022), a 538‐bp fragment of the mtDNA control region was amplified using DLF and DLR primers (Blair et al. 2014), following the protocol described in Garrigue et al. (2022). PCR products were sent for Sanger sequencing to GenoScreen (Lille, France). BioEdit V7.2.5 (Hall 1999) was used to visualize and interpret chromatograms.

To compare dugong populations at the Coral Sea scale, a set of microsatellite markers previously used to genotype Australian dugongs (McGowan et al. 2023) was analyzed. Twenty‐six dugong‐specific microsatellite loci characterized by Broderick et al. (2007) were amplified in eight multiplex PCR reactions as described in Seddon et al. (2014) using the Multiplex PCR kit (Qiagen). Amplified fragments were separated by capillary electrophoresis at the Gentyane Platform (INRAE, Clermont‐Ferrand) on an ABI 3130XL Genetic Analyzer sequencer (Applied Biosystems) with GS‐500‐LIZ (Applied Biosystems) as the internal size marker. Alleles were scored using GeneMapper V. 4.0 (Applied Biosystems). Because Australian and New Caledonian samples were not run side by side, we maximized data comparability by: (1) using the exact same primers as McGowan et al. (2023), generously provided by the authors; and (2) using the exact same bins as for scoring the Australian samples in McGowan et al. (2023), also shared by the authors.

Finally, to assess small scale genetic structure and relatedness at the New Caledonia scale with a higher resolution than with microsatellite markers, single nucleotide polymorphism sites (SNPs) were analyzed. The characterization of genome‐wide SNPs was carried out via ddRAD (double digest restriction site associated DNA; Peterson et al. 2012) sequencing. A library was constructed as described in Daguin‐Thiebaut et al. (2021) using the restriction enzymes PstI and MspI. The quality of the resulting library was controlled using a TapeStation High Sensitivity D1000 screen tape (Agilent Technologies, California) and the library was sent for paired‐end sequencing (2 × 150 bp) on a NovaSeq X Plus sequencing system (Illumina) at Novogene (Munich, Germany).

### 2.3 Data Analysis

#### 2.3.1 Mitochondrial DNA Analysis

Sequence analyses for the mtDNA control region were carried out to assess the contribution of the new data compared with the results presented in Garrigue et al. (2022). For this, the 13 newly generated sequences were analyzed jointly with the 55 New Caledonian sequences published in Garrigue et al. (2022) and 945 sequences retrieved from GenBank (see Figure S1 for references), using Mega V11.0.13 (Kumar et al. 1994). A median‐joining haplotype network was constructed using POPART V1.7.2 (Leigh and Bryant 2015). The number of haplotypes *h*, the number of polymorphic sites *s*, the haplotypic diversity *H*_*d*_, and nucleotide diversity *π* were estimated in ARLEQUIN V3.5.2.2 (Excoffier et al. 2005).

#### 2.3.2 Nuclear Microsatellite Analysis

From the microsatellite genotyping, individuals with more than 50% of missing data were excluded from the dataset. The presence of null alleles was tested and their frequencies estimated using MICRO‐CHECKER V2.2.1 (Van Oosterhout et al. 2004). Allelic richness *A*_*R*_ (based on a minimum sample of 49 individuals, the final number of New Caledonian multilocus genotypes retained for the analyses) was estimated by the *allelic richness* function from the *hierfstat* R package V0.5.11 (Goudet and Jombart 2005); observed heterozygosity, expected heterozygosity, and Wright's *F*_IS_ fixation index were estimated in ARLEQUIN. Deviations from Hardy–Weinberg equilibrium were tested using exact tests in GENEPOP WEB V4.7.5 (Raymond and Rousset 1995). Genotypes of 293 samples from Australia, scored at the same microsatellite loci and available from McGowan et al. (2023) were added prior to conducting the following data analyses. The compatibility of the two datasets was visually estimated by comparing allele frequency distributions obtained from each dataset in order to detect eventual shifts in allele distributions (Figure S2).

The genetic structure at the Coral Sea scale was inferred using several approaches. First, a factorial correspondence analysis (FCA) was made using GENETIX V4.05 (Belkhir et al. 2004). The structure was further analyzed through a Bayesian clustering method implemented in STRUCTURE V2.3.4 (Pritchard et al. 2000) using the “admixture” model, without prior population definition. Individual ancestry probabilities were estimated for each ancestral population K, with K varying from 1 to 10. For each value of K, coefficients were estimated in 10 runs of 100 replicates, for 500,000 iterations with a discarded burnin of 10%. Individual's posterior probabilities of membership to each cluster were summarized using CLUMPAK (Kopelman et al. 2015) and plotted using the R package *ggplot2* V3.5.1 (Wickham 2016). Pairwise *F*_ST_ among regions was estimated in ARLEQUIN, with the significance tested using 1000 permutations.

#### 2.3.3 SNPs Analysis

SNPs genotyping was carried out using the Stacks V2.69 pipeline (Catchen et al. 2013). The *process_radtags* function was used to demultiplex, filter, and exclude low‐quality reads. Reads were aligned to the complete genome of *Dugong dugon* with chromosome‐level assembly available on Genbank at accession number GCA_030035585.1 (Baker et al. 2024) using *bwa‐mem* command‐line tools (Li and Durbin 2009). The *gstacks* function was used to identify SNPs within the meta population for each locus, genotype each individual at each identified SNP, and phase the SNPs at each locus in each individual into a set of loci. Then, the *population* function was used to remove SNPs that were present in too few samples or localities. Individuals for which more than 30% of SNPs had missing values were removed from the data set. Then, SNPs with more than 5% of missing data, a minimum allele frequency below 0.05, and a minimum depth read < 10 were excluded. Finally, SNPs showing linkage disequilibrium were removed (LD > 0.2). Mean observed heterozygosity and mean expected heterozygosity were estimated for the New Caledonian population using the *gl.Ho* and *gl.He* functions in the *dartR* package V2.9.7 (Mijangos et al. 2022). The *basic.stats* function of this same package was used to estimate the inbreeding index *F*_*IS*_, and its significance was tested by permutation of gene copies among individuals within localities.

The presence of genetically related individuals was inferred through parentage analyses using the *sequoia* R package V2.11.2 (Huisman 2017). This package determines parentage by comparing the likelihood ratio of a pair being a parent–offspring duo to the likelihood of the next most‐likely alternative relationship. If not all parents have been genotyped, it identifies clusters of half‐siblings and full‐siblings. Pairwise relatedness among New Caledonian individuals was estimated using Loiselle's kinship coefficient *F*_*ij*_ (Loiselle et al. 1995) with the *kinship_Loiselle* R function from the *RClone* package V1.0.3 (Bailleul et al. 2016). Pairwise relatedness was then statistically compared (1) within locality versus between localities with a Mann–Whitney test to accommodate small sample sizes, and (2) within locality across all sites using a Kruskal–Wallis test followed by a Dunn post hoc test.

The genetic structure at the New Caledonia scale with the SNP genotypes was inferred using different approaches. First, a principal coordinate analysis (PCoA) was performed using Euclidean distances with the *gl.pcoa* R function from the *dartR* package. Then, the population structure was analyzed by a STRUCTURE‐like algorithm implemented in the *snmf* function of the *LEA* R package V3.18.0 (Frichot and François 2015). This function provides least‐squares estimates of ancestry proportion using a sparse non‐negative matrix factorization method, which can handle larger datasets more rapidly than the time‐consuming STRUCTURE software. These admixture proportions were estimated in 10 runs of 100 replicates for each K from 1 to 10. The optimal number of ancestral populations was determined by a cross‐entropy criterion, the best run having the smallest entropy value. To further investigate the accuracy of individuals' assignments, each individual was assigned to a cluster based on its genetic identity by a support vector machine (SVM) classification model implemented in the R package *assignPOP* V1.3.0 (Chen et al. 2018). These assignments were compared with the regions, either North or South (see results), where samples were effectively collected to estimate the accuracy of the assignment. This function uses a Monte Carlo cross‐validation procedure to estimate the mean and variance of assignment accuracy by subsampling random individuals from the entire dataset to form a training dataset containing 50%, 70%, or 90% of the individuals and using the top 10%, 25%, 50% or 100% of loci showing the highest *F*_ST_ values, compared to all loci. Subsampling was repeated 1000 times for each proportion of training individual and loci combination. Model performance was assessed by assigning an origin to individuals from the test dataset (i.e., individuals that were not included in the training dataset) and calculating the percentage of accuracy of these assignments.

Pairwise‐*F*_ST_ was estimated among localities and tested for significance using 1000 permutations in the *gl.fst.pop* function of the R package *dartR*. To investigate the connectivity among sites and between subpopulations, contemporary migration rates were estimated in BA3SNPs V3.0 (Mussmann et al. 2019). MCMC was run for 10,000,000 iterations with a discarded burn‐in of 10% and an interval of 1000 permutations between samples. For the first analysis, mixing parameters for allele frequencies and migration rates were set to 0.5 and 0.4 to decrease the acceptance rate and avoid inappropriate mixing of the MCMC. For the second one, these parameters were set to 0.7 and 0.4. The convergence of the chain was checked in Tracer V1.7.2 (Rambaut et al. 2018).

The effective size of the New Caledonian population was estimated using two approaches: ONeSAMP 3.0 (Hong et al. 2024) and NeEstimator 2.1 (Do et al. 2014). The first algorithm uses an Approximate Bayesian Computation method to estimate effective size on SNPs data and is quite appropriate for small samples. The second software estimates contemporary effective population size on multilocus diploid genotypes, using a method based on linkage disequilibrium. Since the generation time of dugongs is 22 to 25 years (Brownell et al. 2019), only the 18 adults collected between 2011 and 2021 were retained for these analyses in order to limit the estimation to individuals potentially belonging to a single generation. This calculation was performed by 5000 trials. Ratios between effective population size and census size were estimated using mean effective population size *N*_*e*_ for both lower and higher census size estimates, based on Hagihara et al. (2018) for the year 2012.

## 3 Results

### 3.1 Genetic Diversity

Of the 13 post‐2020 collected individuals, 12 were successfully amplified for mtDNA. These newly generated mtDNA sequences were strictly identical to the most common haplotype (DduNC01) previously found in New Caledonia (Garrigue et al. 2022), and, with their addition to the 53‐sequences previous dataset, the sequence data analysis revealed that 97% of the New Caledonian dugong population shared this same haplotype. Haplotypes DduNC02 and DduNC03 were represented by only one individual (approximately 1.5% of the population) from the Noumea region. Estimated haplotypic and nucleotide diversities were consequently very low at the New Caledonia level (*H*_*d*_ = 0.0611 ± 0.0409 and *π* = 0.0001 ± 0.0002; Table 1), and across all localities, with null values except for Noumea (*H*_*d*_ = 0.1857 ± 0.1102 and *π* = 0.0003 ± 0.0005). The newly produced median‐joining haplotype network (Figure S1) was thus similar to the one presented in Garrigue et al. (2022), with the three New Caledonian haplotypes being imbedded within the so‐called “Widespread” Australian lineage.

Among the 26 amplified microsatellite loci, 18 produced interpretable patterns for New Caledonian dugongs and were further kept for scoring. Loci DduC03, DduC09, DduE03, DduE08, and DduD08 were monomorphic or prone to null alleles and were excluded from the data analyses. Nineteen individuals from New Caledonia were excluded due to high proportions (> 50%) of missing genotypes. The final microsatellite dataset was thus composed of 342 samples (*N* = 49 in New Caledonia; *N* = 293 in Australia) genotyped at 13 loci, with 2 to 5 alleles per locus for New Caledonian samples (Figure S2). All alleles found in New Caledonia were present in Australian samples, and from the comparison of allele frequency distributions, only one locus out of the 13 analyzed, DduE03, showed a 2 bp shift in the distribution that may potentially inflate pairwise‐*F*_ST_ estimates (Figure S2). Over all New Caledonian samples, the estimated allelic richness was 2.472, the observed heterozygosity was 0.401 ± 0.260, and the expected heterozygosity was 0.427 ± 0.285. These indices were considerably lower than those found in Northern (*A*_*R*_ = 7.11 ± 2.77; *H*_*o*_ = 0.689 ± 0.170; *H*_*e*_ = 0.725 ± 0.152) and Southern Queensland (*A*_*R*_ = 5.09 ± 1.91; *H*_*o*_ = 0.596 ± 0.191; *H*_*e*_ = 0.601 ± 0.201). Within New Caledonia, the *F*_IS_ estimated from the microsatellite data was negative and not significant (*F*_IS_ = −0.030, *p* = 0.707, Table 1). Within the five New Caledonian localities, the range of each parameter was 1.266 ± 0.411 to 1.354 ± 0.295 for the allelic richness, 0.265 ± 0.245 to 0.378 ± 0.328 for the observed heterozygosity, and 0.111 ± 0.220 to 0.352 ± 0.293 for the expected heterozygosity. Bourail was the only locality exhibiting significant inbreeding (*F*_IS_ = 0.195, *p* = 0.021) at the microsatellite loci.

From the ddRAD library, 231.8 GB of raw sequence data and 1,363,735,380 reads were generated from the sequencing. The mean number of reads per sample was 15,776,492, ranging from 8840 to 75,000,522 per sample. The final dataset included 2499 SNPs genotyped across 48 individuals, with 2.92% of missing data. Overall samples, the average observed heterozygosity for all SNPs was 0.337, while the expected heterozygosity was 0.341. The *F*_IS_ inbreeding index of the overall New Caledonian samples was 0.023 and was found to be significant (*p* < 0.001). Within localities, observed heterozygosities ranged from 0.324 in Bourail to 0.369 in Poum. Significant *F*_IS_ were found in Voh, Bourail, and Noumea, with *F*_IS_ estimates ranging from 0.002 in Noumea (*p* = 0.037) to 0.021 in Bourail (*p* < 0.001, Table 1).

### 3.2 Population Structure Analysis

**FIGURE 2:**
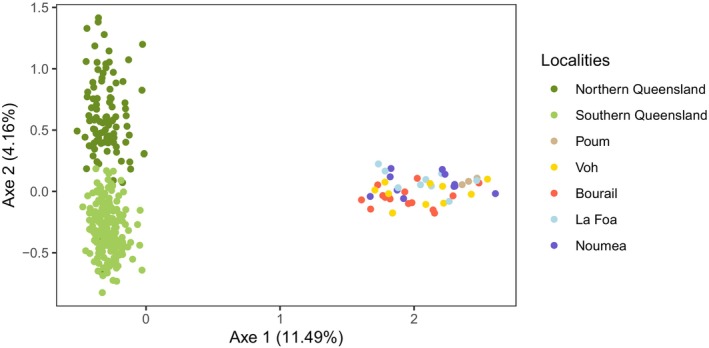
Factorial correspondence analysis (FCA) based on dugong genotypes obtained from the analysis of 13 microsatellite loci. The geographical origin of each sample is indicated by different colors.

The FCA based on all individual microsatellite genotypes from New Caledonia and Australia revealed a clear separation in three populations using the first two axes (Figure 2): The first axis separated the New Caledonian population from the Australian populations, and the second axis disentangled the two Queensland populations, referred to as the north and south clusters by McGowan et al. (2023). This was also clearly illustrated in the clustering analysis of the dugongs in the Coral Sea, showing a number of ancestral populations best fitting the data of *K* = 2 (Figure S3A). All dugongs from New Caledonia grouped into a first cluster with a mean assignment proportion of 0.997, while Australian dugongs were found in a second cluster, with similarly high assignment proportions (mean admixture coefficient of 0.996). When assuming three ancestral populations (*K* = 3, Figure S3B), the Australian cluster was further divided into two subclusters, each comprising individuals from the eastern Queensland coast, located north and south of the known genetic break, respectively, with a slight admixture. Consistently, the dugongs of New Caledonia exhibited high genetic differentiation from Australian dugongs of the northern and southern clusters (*F*_ST_ = 0.388, *p* < 0.001 and *F*_ST_ = 0.425, *p* < 0.001, respectively), while *F*_ST_ was more than four times lower between the two Australian subpopulations (*F*_ST_ = 0.092, *p* < 0.001).

**FIGURE 3:**
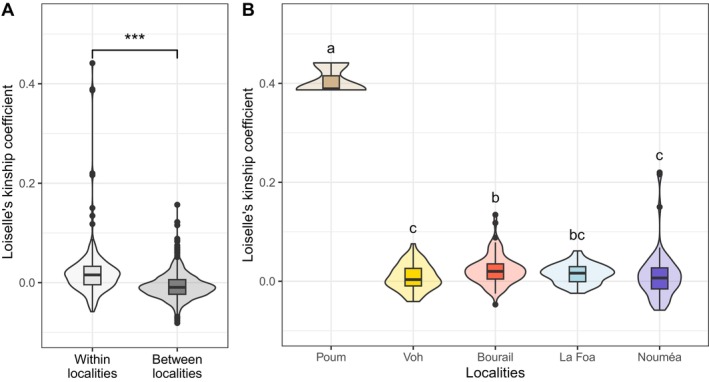
Distribution of pairwise relatedness (Loiselle's kinship coefficient *F*_*ij*_) among New Caledonian dugongs estimated from genome wide SNPs (A) between individuals sampled in the same localities as compared to different localities, (B) between individuals within each locality. Letters at the top of each violin plot represent the significance of differences among localities.

Within New Caledonia, individuals sampled within the same locality showed significantly higher mean pairwise relatedness based on genome‐wide SNPs (mean *F*_*ij*_ = 0.021 ± 0.055) than individuals taken from different localities (mean *F*_*ij*_ = −0.007 ± 0.025; Mann–Whitney test: *p* < 2.2e‐16, *W* = 321,738) (Figure 3A). Within each locality, the highest pairwise relatedness (mean *F*_*ij*_ = 0.406 ± 0.030) was found for Poum (Figure 3B) and was attributed to the presence of parent‐offspring couples detected by the parentage analysis (with pairwise relatedness of 0.441 between individuals DduNC12‐158 and DduNC13‐163; 0.389 between individuals DduNC12‐158 and DduNC13‐164, and 0.386 between individuals DduNC13‐163 and DduNC13‐164). Individual DduNC12‐158 (2 m long female) was found stranded in 2012 by a professional fisher, with marks suggesting that it had been intentionally killed. Samples from DduNC13‐163 and DduNC13‐164 (unknown sex and size) were obtained from an illegal poaching seizure in 2013. The locality of Bourail showed the second highest mean pairwise relatedness (mean *F*_*ij*_ = 0.023 ± 0.030), followed by La Foa (mean *F*_*ij*_ = 0.015 ± 0.019), Noumea (mean *F*_*ij*_ = 0.012 ± 0.050), and Voh (mean *F*_*ij*_ = 0.008 ± 0.026). Differences in mean *F*_*ij*_ were significant between Poum and all other localities, as well as between Bourail and both Voh and Noumea (Figure 3B; Kruskall–Wallis test: *H* = 19.995, *p* = 0.0005).

**FIGURE 4:**
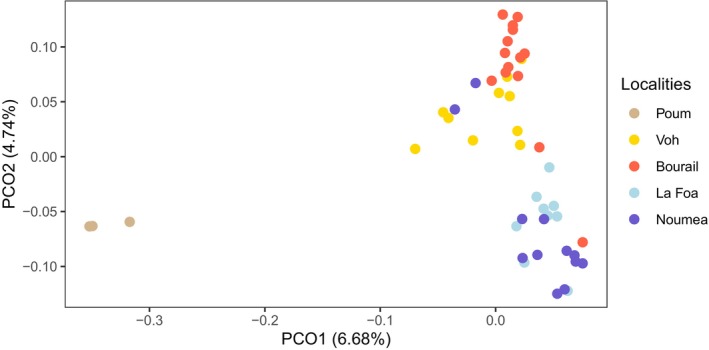
Genetic differentiation of dugongs across New Caledonia. Principal coordinate analysis (PCoA) based on 2499 SNPs showing New Caledonian dugongs color‐coded based on the five localities near which they were sampled.

Within New Caledonia, the first axis of the PCoA conducted on genome‐wide SNPs separated the samples of Poum from the rest of the individuals, which were more or less organized on the second axis according to their geographic position along a north–south gradient (Figure 4). This result translated into a strong genetic differentiation at the scale of New Caledonia (Table 2), with pairwise‐*F*_ST_ estimates ranging from 0.002 (*p* = 0.092, between Noumea and La Foa) to 0.198 (*p* < 0.001, between Poum and La Foa). Poum appeared highly differentiated from all other four localities, a result likely inflated by the presence of related individuals within the Poum sample.

**TABLE 2:** Pairwise *F*_*ST*_ values estimated between sampling localities in New Caledonia, based on the analysis of 2499 SNPs.

	Poum	Voh	Bourail	La Foa
Voh	0.1645***			
Bourail	0.1828***	0.0185***		
La Foa	0.1980***	0.0258***	0.0294***	
Noumea	0.1874***	0.0254***	0.0366***	0.002

*** *p* < 0.001.

**FIGURE 5:**
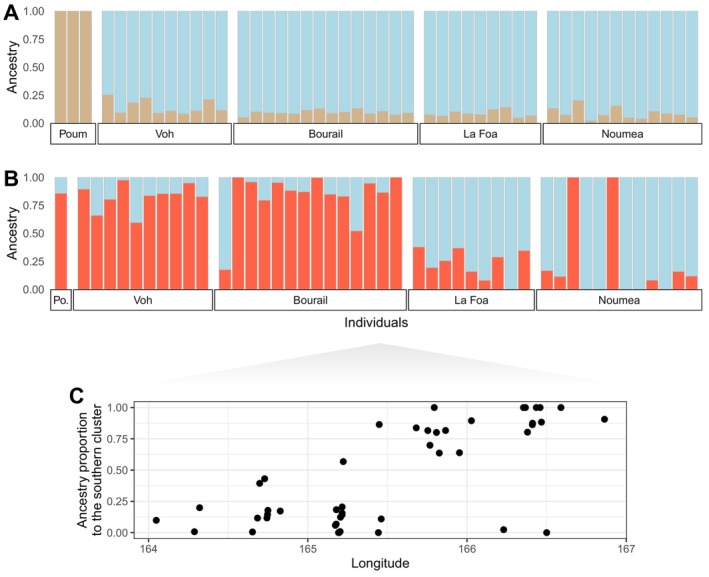
Individual admixture coefficients based on SNPs data (A) from the global population to the different clusters for a number of ancestral populations *K* = 2 best fitting the data according to the snmf function; (B) from the population to the different clusters for a number of ancestral populations *K* = 2 while excluding 2 individuals from Poum (individuals are sorted by sampling site from north to the left to south to the right); (C) individual ancestry proportion of individuals to the southern cluster in relation to the longitude of their sampling sites (from Table S1).

Within New Caledonia, the *snmf* ancestry reconstruction based on genome‐wide SNPs pointed to the likelihood of *K* = 2 ancestral populations (Figure 5A). The individuals were grouped based on their highest ancestry coefficients. Poum individuals clustered together, while all the other samples formed the second cluster. Assuming *K* = 3 ancestral populations (Figure S4), this second cluster was further divided into two clusters strongly discriminating northern and southern individuals. Twenty‐three out of 24 northernmost individuals from Voh and Bourail clustered together (with two individuals from Noumea), while 19 out of 21 southernmost individuals from La Foa and Noumea formed a second cluster (with one individual from Bourail). Because the relatedness found among the three individuals of Poum could bias population structure analyses, two samples from this locality were randomly removed and the *snmf* analysis was rerun. While the number of ancestral populations best fitting the data was then *K* = 1, the entropy value observed for a number of clusters *K* = 2 (0.860) was very close to the minimum entropy value for *K* = 1 (0.849, Figure S5). For *K* = 2 (Figure 5B), two admixed clusters could be identified, within varying degrees of admixture among localities: individuals from Bourail, Voh and the individual from Poum (i.e., the northernmost samples) had a mean admixture proportion of 0.826 to cluster 1, while individuals from La Foa and Noumea (the southernmost samples) had a mean admixture proportion of 0.785 to cluster 2. This pattern was clearly shown when plotting the distribution of admixture coefficients obtained by *snmf* as a function of the longitude of each individual's sampling locality (Figure 5C). Few exceptions were found, with two individuals sampled around Noumea (one male and one female, both adults) having higher admixture proportions for the northern cluster (~0.99), and one male sampled in Bourail having a higher admixture proportion to the southern cluster (~0.82). When the individuals were assigned to one of these two clusters based on their highest admixture coefficient, the estimated *F*_ST_ between the northern and southern clusters was 0.026 (*p* < 0.001). Estimated migration rates were found to be asymmetric between the northern and southern clusters, with the southbound migration rate estimated at *m* = 0.100 ± 0.028 and the northbound migration rate estimated at *m* = 0.220 ± 0.035.

The assignment accuracy test showed that the proportion of loci used to build the model had a significant effect on the accuracy of assignments for both northern (Pearson correlation test, *t* = −6.1977, *p* = 0.025) and southern clusters (Pearson correlation test, *t* = 6.053, *p* = 0.026) when using 50% of training individuals. Accuracy increased consistently with a higher proportion of loci for the southern cluster and decreased for the northern cluster (Figure S6). The proportion of training individuals had no effect on the accuracy of assignment for the northern subpopulation, while there were significant differences in accuracy when using 50% or 90% of the individuals for the southern cluster (ANOVA, *F* = 7.17, *p* = 0.002). For the southern cluster, a minimum mean accuracy of 0.7967 was found when using 50% of training individuals with the top 10% of higher *F*_ST_ loci. A larger proportion of training individuals (90%) yielded higher accuracy, peaking at 0.9085 for top 25% loci. The lowest accuracy for the northern subpopulation of 0.922 was found when using 0.5 with all loci. The model was more accurate (0.950) with 90% of trained individuals and 50% of higher *F*_ST_ loci.

### 3.3 Effective Population Size

Based on the SNP dataset, the effective population size was estimated to be *N*_*e*_ = 130 breeding individuals using ONeSAMP, with a lower 95% quantile of 106 and a higher of 160 (Table 3). NeEstimator yielded lower estimates: The effective population size was estimated at Ne = 99 individuals, with a 95% confidence interval ranging from 95 to 103. Based on a census size ranging from 792 to 1166 dugongs estimated by aerial monitoring (Cleguer et al. 2017; revised Hagihara et al. 2018), the ratio between the effective population size and its census size was estimated to vary between 0.164 and 0.111 using ONeSAMP estimates, and 0.125 and 0.085 using NeEstimator estimates.

**TABLE 3:** Effective population size estimates (with lower and upper confidence intervals) of New Caledonian dugongs based on the analysis of 2499 SNPs using two methods (ONeSAMP and NeEstimator).

	Overall		Northern cluster		Southern cluster	
	ONeSAMP	NeEstimator	ONeSAMP	NeEstimator	ONeSAMP	NeEstimator
Lower CI	105.3	95.1	156.1	121.5	169.2	111.0
Ne estimate	129.8	98.7	205.5	136.7	225.6	124.9
Upper CI	159.3	102.6	258.5	156.1	278.3	142.8

*Note:* Estimates are also given for the northern and southern clusters.

## 4 Discussion

By utilizing multiple genetic markers and an expanded sampling of tissue samples, we enhanced the characterization of the small, endangered dugong population of New Caledonia. Our main findings confirmed the extremely low genetic diversity in this population, revealed a strong genetic divergence between New Caledonian and Australian populations, and provided evidence of substructuring within New Caledonia. In addition, we found that the effective size of the New Caledonian dugong population is particularly low.

### 4.1 Genetic Erosion and Emerging Signs of Inbreeding

The genetic diversity of the dugong population in New Caledonia is exceptionally low, irrespective of the genetic marker used. Indeed, with a larger sample size, our findings confirm the extremely low mitochondrial diversity observed by Garrigue et al. (2022), further emphasizing that the New Caledonian population has the lowest haplotypic and nucleotide diversity recorded across the species' range. Across dugong populations, haplotypic diversity typically ranges from 0.33 to 1, and nucleotide diversity from 0.010 to 0.096, depending on the region (Garrigue et al. 2022). In contrast, the New Caledonian population exhibits estimates of 0.059 and 0.0001, respectively. Additionally, the nuclear allelic richness of the New Caledonian population, based on the analysis of 13 microsatellite markers, was found to be two to three times lower than that of Australian populations. Observed and expected heterozygosities, based on microsatellite loci, are also nearly half of what is observed in other dugong populations (Poommouang et al. 2022; McGowan et al. 2023; Seddon et al. 2014).

While no evidence of inbreeding was detected at the microsatellite loci, high‐resolution SNP data revealed evidence of inbreeding at the scale of the New Caledonian dugong population. The significant inbreeding coefficient (*F*_IS_ = 0.023) derived from SNP data aligns with values reported for other sirenian populations, including distinct dugong populations along the eastern Queensland coast of Australia (*F*_IS_ = 0.015–0.038; Seddon et al. 2014), dugong populations in Thailand (*F*_IS_ = 0.055; Bushell 2013), as well as manatee populations in Florida (*F*_IS_ = 0.027–0.046; Tucker et al. 2012) and Belize (*F*_IS_ = 0.012; Hunter et al. 2010). These findings indicate moderate levels of inbreeding, which are characteristic of small and isolated populations. The critical combination of extremely low genetic diversity and significant inbreeding highlights the fragile state of the New Caledonian dugong population and its likely limited resilience to environmental and anthropogenic pressures.

### 4.2 A Precarious Genetic Status

In New Caledonia, the dominance of a single haplotype in 97% of the individuals, differing by only one base pair from the two remaining haplotypes, supports the hypothesis proposed by Garrigue et al. (2022), suggesting that the New Caledonian population may have originated from a migration event involving a very small number of dugongs. Consistent with this hypothesis, our findings reveal a small effective population size (*Ne*) of 95 to 160 individuals for New Caledonian dugongs (including lower and upper bounds based on two distinct methods that provided very similar small estimates). Sampling design and SNP filtering can substantially influence estimates of effective population size (Marandel et al. 2020). Key factors affecting these estimates include sample size, minimum allele frequency (MAF), and the distribution of missing data, particularly when it is nonrandom. In our study, the dataset comprised a large number (∼2500) of SNPs, applied a moderate MAF threshold (0.05), and had a very low proportion of missing data (< 5% per SNP). However, the relatively small sampling size (*N* = 18) used intentionally (to avoid mixing individuals from different generations) likely had the greatest impact on Ne estimation. Nevertheless, the 95% confidence interval bounds for *Ne* were close to the mean, indicating that the estimates are likely robust and reliable.

For comparison, Bilgmann et al. (2021) estimated that the effective population size of the Australian sea lion, ranging between 160 and 424 breeding individuals, is insufficient to maintain robust genetic variability. Similarly, Runge et al. (2017) suggested that an effective population size of fewer than 500 individuals represents a near‐extinction threshold for the Florida manatee, a species closely related to dugongs. We also found a low *N*_*e*_‐to‐census size ratio, with the caveat that *N*_*e*_ focuses on adult mature individuals, while the census size estimated through aerial surveys encompasses all age classes (Cleguer et al. 2017). Effective population size is typically much smaller than census size (Gagne et al. 2018), and the ratios for the New Caledonian population are comparable to those reported for neighboring Australian populations, which range from 0.062 to 0.129 depending on the site. The estimated effective population size of the New Caledonian dugongs falls below these critical thresholds, underscoring the endangered status of this small population. These results further highlight the precarious genetic state of the population.

### 4.3 The New Caledonian Dugong: A Genetically Isolated Population

At the scale of the Coral Sea, dugongs appear to be divided into two genetically distinct populations. The two Australian subpopulations from Queensland, while significantly differentiated (*F*_ST_ = 0.092 in this study using a subset of individuals; *F*_ST_ = 0.011 in McGowan et al. 2023), still exhibit gene flow. This is evident from the presence of individuals assigned to each genetic cluster within the other subpopulation. In contrast, the New Caledonian population is highly differentiated from the Australian populations (*F*_ST_ = 0.388–0.425), with no individuals assigned to Australian genetic clusters in New Caledonia, and vice versa.

The ability to detect the two known genetic clusters within the Australian populations suggests that the subset of microsatellite markers used in this study (13 out of 22 loci from McGowan et al. 2023) provided sufficient resolution to identify subtle genetic differentiation. However, a key limitation in interpreting population structure from microsatellite data is the lack of calibration between the Australian and New Caledonian samples. Specifically, the Australian DNA samples were not amplified and genotyped simultaneously with the New Caledonian samples, which may have introduced sequencing and/or scoring errors. We acknowledge that this could potentially bias the estimates of genetic differentiation. To mitigate this risk, we employed the same primers, PCR multiplexes, and allele binset (a set of expected allele size ranges for each locus) as in McGowan et al. (2023) to ensure consistency in genotyping the New Caledonian samples. Moreover, an examination of allele frequency distributions revealed no major discrepancies between the two regions. Most importantly, our findings are consistent with those of Oremus et al. (2015) who reported substantial genetic differentiation between Australian and New Caledonian dugong populations. In their study, 10 of the 13 loci used here were genotyped on 200 samples (33 from New Caledonia included in this study, 167 from Australia), of which 31 (16 from New Caledonia and 15 from Australia) were initially amplified and genotyped side by side in the laboratory of Associate Professor Jennifer Seddon at the University of Queensland in order to calibrate the two datasets. Their analyses revealed that all dugongs from New Caledonia grouped into a distinct genetic cluster, with admixture coefficients close to one, and showed high levels of differentiation from Australian populations (pairwise *F*_ST_ values ranging from 0.230 to 0.407, Oremus et al. 2015). These findings are in line with the clear genetic structure observed in our study, which included a larger sample size for both populations and additional microsatellite loci.

Besides, the large number of loci containing null alleles—excluded from our analysis—suggests that primer mispairing due to mutations in the flanking regions of microsatellites likely contributed to these discrepancies. This observation further supports the strong genetic divergence estimated between the New Caledonian and Australian populations. To obtain a more precise understanding of the level of differentiation, an integrative analysis should be conducted using a broader set of appropriate markers and samples processed simultaneously. Such an approach would provide a clearer picture of the genetic isolation between these two populations.

In conclusion, our results suggest a complete lack of gene flow between the New Caledonian and Australian populations. This is consistent with the significant geographic separation (1330 km) between New Caledonia and Australia and the fact that dugongs very rarely migrate long distances across open waters (Hamylton et al. 2012). Consequently, natural recolonization of New Caledonia by the Australian population following a potential mass mortality event is unlikely. While isolation from the Australian population is now well documented, the potential for connectivity with other Melanesian populations, such as those in Vanuatu, the Solomon Islands, and Papua New Guinea, remains to be investigated. However, substantial gene flow between these locations is likely highly restricted, if not improbable, given the approximately 350 km of deep water (> 2000 m) separating New Caledonia from its nearest neighbor, Vanuatu.

### 4.4 Evidence of Genetic Structure and Limited Gene Flow at the New Caledonia Scale

Our analyses revealed further significant substructuring within the New Caledonian dugong population. Individuals sampled within the same locality exhibited significantly higher mean pairwise relatedness, which translated into notable genetic differentiation between nearly all sampling localities. This pattern was further supported by the PCoA analysis, where geographically remote localities appeared more genetically distinct than closer ones, reflecting New Caledonia's geographic layout. The higher kinship coefficients observed within localities compared to between localities suggest a predominance of local reproduction and limited gene flow between locations. This finding aligns with the average dugong movement distances, which rarely exceed 100 km, including evidence from New Caledonia (Sheppard et al. 2006; Cleguer et al. 2020; Derville et al. 2022). Similar patterns of genetic isolation between distant sites have also been observed in Australian dugong populations (Seddon et al. 2014; McGowan et al. 2023), emphasizing the role of restricted movement in shaping genetic structure.

Dugongs are generally described as solitary animals, unlike other marine mammals, such as odontocete populations where genetic structure can be strongly influenced by social behavior (e.g., the Indian Ocean bottlenose dolphin *Tursiops aduncus* , Ansmann et al. 2012). The mating behavior of dugongs—specifically where and when it occurs—remains a significant knowledge gap in New Caledonia, as it does throughout their range (Infantes et al. 2020; Adulyanukosol et al. 2007; Anderson and Barclay 1997). Yet, the relatively sedentary nature of dugongs suggests that mating likely occurs within localized areas, potentially contributing to the formation of fine‐scale population structure. In addition, in New Caledonia, seagrass habitats are patchily distributed over a wide lagoon, with large variation in density and quality across space (Andréfouet et al. 2021) so that dugongs' movements and population structure are likely influenced by the distribution of these meadows (Cleguer et al. 2020; Derville et al. 2022) in addition to the influence of environmental factors such as tides and water temperature (Cleguer et al. 2024).

Our analysis also identified specific instances where localized kinship may have influenced genetic structure results. For example, the three individuals sampled in Poum form a highly differentiated genetic cluster with the highest *F*_ST_ values. The fact that Poum samples exhibit high pairwise Loiselle's coefficients (0.386–0.441) indicates that these individuals likely belong to the same family, being closely related. These three samples all came from meat retrieved from poaching events (i.e., two samples in 2013 from meat conserved in two different freezers, one sample from 2012 retrieved from a fisher). It is possible that these individuals include poached mother–calf pairs, which could skew the overall population structure analysis. An alternative, though not mutually exclusive, explanation for the strong genetic differentiation observed in the Poum samples is that this locality has experienced true differentiation, potentially driven by intensified poaching pressure and/or its geographic isolation at the northernmost tip of New Caledonia's main island. To more accurately assess the genetic structure of the population, additional samples from Poum and other northern or northeastern regions are needed to determine whether the observed structure reflects real population divergence or is simply an artifact of close kinship among a small number of individuals. Nevertheless, Nazareno et al. (2017) demonstrated that reliable *F*_ST_ estimates can be obtained from as few as two individuals when using ddRADseq data, provided the dataset includes a sufficiently large number of SNPs (i.e., > 1500). For all other localities in this study—each represented by 9 to 14 individuals and genotyped at a large number of markers—the *F*_ST_ estimates can therefore be considered sufficiently robust for interpretation.

### 4.5 Identification of Two Environmentally Driven Genetic Clusters

At the New Caledonia scale, high‐resolution genetic markers revealed two distinct genetic clusters, with strong individual memberships based on geographic location. The relatively low levels of mixed ancestry and the small number of dugongs with cluster assignments opposite to their geographic location suggest low levels of movement between Bourail and La Foa (with a breakpoint located just south of the Bourail bay). These results are consistent with past satellite‐tracking data that showed no movement of northern individuals to the southern region and vice versa based on the tracking of 16 individuals along the west coast of New Caledonia (Cleguer et al. 2020; Derville et al. 2022).

In contrast to species where social behavior strongly influences spatial distribution (e.g., Svendsen 1974; Wirsing et al. 2007), the genetic differentiation in dugongs is more likely linked to environmental factors. Environmental barriers, such as the characteristics of Bourail Bay, may contribute to the observed genetic break. Bourail Bay is exposed to the open sea, deep, and wide, potentially hindering dugong movement across this region. Such environmental contrasts could create a cryptic barrier between northern and southern clusters. Predation risk is also likely to influence the distribution of dugongs in this area, which is known to be frequented by large sharks, as evidenced by documented tiger shark attacks on humans (Maillaud et al. 2022).

Despite this genetic division, our analysis identified three putative migrants between the two clusters, consistent with low but significant estimated asymmetric gene flow. However, one of these individuals, initially assigned to the southern cluster, was sampled from a floating carcass in Bourail. Given the possibility that it drifted from the south, its classification as a migrant may be inaccurate. Our genetic findings suggest that dugongs may occasionally traverse Bourail Bay, though such movements are likely rare and more frequently occur from south to north.

### 4.6 Management Implications

Marine conservation translocations are increasingly common worldwide (Swan et al. 2016). These efforts aim to support population restoration by either reinforcing critically endangered populations or reintroducing species to regions where they were once extirpated. However, several cases highlight the significant challenges associated with marine mammal translocations, including habitat suitability, predation, human threats, and behavioral adaptation. As a result, their success has often been variable or limited (e.g., Hawaiian monk seals ( *Neomonachus schauinslandi* ) in Northwestern Hawaiian Islands, Baker et al. 2011; sea otters ( *Enhydra lutris* ) in Oregon, USA, Jameson et al. 1982; the Antillean subspecies of the West Indian manatee ( *Trichechus manatus manatus* ) in Brazil, Normande et al. 2015; the Yangtze River dolphin or baiji ( *Lipotes vexillifer* ) in China, Turvey et al. 2007).

Our study highlights the small effective population size and significant inbreeding within the New Caledonian dugong population. However, we emphasize the substantial costs and risks associated with translocation efforts. In the case of New Caledonia, we do not recommend translocation, as it carries a high risk of introducing new diseases or parasites, which could, at best, exacerbate the population's challenges and, at worst, trigger a mass mortality event.

Despite these concerns, there is still hope for the recovery of New Caledonia's dugong population if appropriate management measures are implemented. Along the western coast of Grande Terre, we suggest that the two genetic clusters—likely corresponding to two breeding units, north and south of Bourail Bay—should be considered when designing monitoring and management strategies. Given that dugong movements across this region are rare, conservation plans should recognize these distinct population structures. Additionally, since environmental management falls under provincial jurisdiction, it is crucial for New Caledonia's North and South Provinces to integrate these management units into their respective dugong conservation strategies.

## Author Contributions

**Paolo Verger:** data curation (equal), formal analysis (equal), investigation (equal), writing – original draft (equal), writing – review and editing (equal). **Claire Garrigue:** conceptualization (equal), funding acquisition (equal), project administration (equal), writing – review and editing (equal). **Claire Daisy Bonneville:** data curation (equal), writing – review and editing (equal). **Solène Derville:** conceptualization (equal), funding acquisition (equal), project administration (equal), writing – review and editing (equal). **Marc Oremus:** conceptualization (equal), data curation (equal), writing – review and editing (equal). **Camille Sant:** data curation (equal), investigation (equal), writing – review and editing (equal). **Cécile Fauvelot:** conceptualization (equal), formal analysis (equal), supervision (equal), validation (equal), writing – original draft (equal), writing – review and editing (equal).

## Disclosure

Benefit‐sharing statement: This project is a national collaboration that includes scientists based in New Caledonia, where the study took place. All local collaborators have been appropriately acknowledged as coauthors. The findings have been communicated to the provincial environmental authorities who coordinate the stranding monitoring network in New Caledonia, supporting local conservation efforts. This work addresses a key conservation priority for dugongs in the region. More broadly, our research group is committed to fostering equitable international scientific partnerships and contributing to institutional capacity building.

## Ethics Statement

All permits required to sample carcasses of dugongs or get biopsy on living animals were obtained from New Caledonian authorities. Handling of live animals was reviewed and approved by New Caledonia's North Province Department of Economic Development and Environment (Permit n°609011‐52), as well as South Province Department of Environment (Permits 3616‐2011/ARR/DENV and 3157‐2012/ARR/DENV) both in charge of reviewing the ethics of animal handlings project within their territory. Animal handling was carried out in respect to relevant guidelines and regulations.

## Conflicts of Interest

The authors declare no conflicts of interest.

## Supporting information


**Data S1:** ece372168‐sup‐0001‐Supinfo.docx.

## Data Availability

DNA sequences are available in NCBI under GenBank accession numbers ON227090 to ON227092. Metadata, individual genotype data for both microsatellite and SNP loci, are available on the EU Open Research Zenodo Repository (https://doi.org/10.5281/zenodo.17047750).
