# Supplementary material for: Unveiling the Genetic Structure of New Caledonian Dugongs Using a Multiscale Genetic Approach: Conservation Challenges for an Isolated Population
Source: Ecol Evol. 2025 Sep 14;15(9):e72168. doi: 10.1002/ece3.72168 (PMC12434184; doi:10.1002/ece3.72168)
Supplement: Supplementary file 1 — Data S1: ece372168‐sup‐0001‐Supinfo.docx. [file ECE3-15-e72168-s001.docx]

**SUPPLEMENTARY INFORMATIONS**

**Unveiling the genetic structure of New Caledonian dugongs using a multi-scale genetic approach: conservation challenges for an isolated population**

**Authors** : Paolo VERGER, Claire GARRIGUE, Claire Daisy BONNEVILLE, Solène DERVILLE, Marc OREMUS, Camille SANT, Cécile FAUVELOT

**Table S1** : Samples description of all dugong analysed in the present study. **Ref_sample** : the code of each sample (DduNCYY-XXX) ; **mtDNA** : mtDNA haplotype availability ; **msats** : microsatellites genotypes availability ; **SNPs** : SNP genotypes availability ; **Location** : location of collection ; **Type Collection** : type of collection (Stranding, biopsy, hunting) ; **Date** : date of collection (JJ:MM:YYYY) ; **Latitude** : latitude in decimal degrees WGS84 ; **Longitude** : longitude in decimal degrees WGS84 ; **Length** : total length in centimeter ; **Sex visual** : sex identified in the field ; **Genetic Sexing** : sex identified by molecular analysis ; **mtDNA haplotype** : haplotypes following Garrigue et al 2022 ; **GenBank Accession** : GenBank accession number ; **Garrigue et al. 2022** : samples used in Garrigue et al. (2022), mtDNA analysis ; **Oremus et al. 2015** : samples used in Oremus et al. (2015), microsatellite data analysis.

| **Ref_sample** | **mtDNA** | **msats** | **SNPs** | **Location** | **Type Collection** | **Date** | **Latitude** | **Longitude** | **Length** | **Sex visual** | **Genetic Sexing** | **mtDNA haplotype** | **GenBank Accession** | **Garrigue et al. 2022** | **Oremus et al. 2015** |
| --- | --- | --- | --- | --- | --- | --- | --- | --- | --- | --- | --- | --- | --- | --- | --- |
| DduNC03-002 | Yes | Yes | Yes | Noumea | BIOPSY | 17/05/2003 | -22,323566 | 166,41015 | NA | NA | M | DduNC01 | ON227090 | Yes | Yes |
| DduNC03-003 | Yes | Yes | Yes | Noumea | BIOPSY | 17/05/2003 | -22,315283 | 166,4112 | NA | NA | F | DduNC01 | ON227090 | Yes | Yes |
| DduNC04-053 | Yes | No | No | Voh | STRANDING | 12/12/2004 | -21,004234 | 164,672358 | ≈250 | M | M | DduNC01 | ON227090 | Yes | Yes |
| DduNC06-002 | Yes | No | No | Noumea | STRANDING | 11/01/2006 | -22,283326 | 166,473828 | 182 | M | M | DduNC01 | ON227090 | Yes | Yes |
| DduNC06-170 | Yes | Yes | Yes | Noumea | STRANDING | 25/10/2006 | -22,239849 | 166,499906 | 254 | M | M | DduNC01 | ON227090 | Yes | Yes |
| DduNC07-161 | Yes | Yes | Yes | Noumea | STRANDING | 02/09/2007 | -22,242955 | 166,434755 | 260 | M | M | DduNC01 | ON227090 | Yes | Yes |
| DduNC08-169 | Yes | Yes | Yes | La Foa | HUNTING | ND | -21,855126 | 165,807896 | NA | NA | M | DduNC01 | ON227090 | Yes | Yes |
| DduNC09-010 | Yes | Yes | Yes | Noumea | STRANDING | 19/01/2009 | -22,30951 | 166,458711 | 283 | F | F | DduNC01 | ON227090 | Yes | Yes |
| DduNC10-002 | Yes | No | No | Bourail | HUNTING | 23/01/2010 | -21,480858 | 165,169036 | NA | M | M | DduNC01 | ON227090 | Yes | Yes |
| DduNC10-009 | Yes | No | No | Bourail | STRANDING | 19/06/2010 | -21,654875 | 165,472105 | NA | NA | NA | DduNC01 | ON227090 | Yes | Yes |
| DduNC11-017 | Yes | Yes | Yes | Bourail | STRANDING | 13/04/2011 | -21,647688 | 165,448501 | ≈120 | M | M | DduNC01 | ON227090 | Yes | Yes |
| DduNC11-265 | Yes | Yes | Yes | Voh | STRANDING | 07/12/2011 | -21,00183 | 164,684308 | 116 | M | M | DduNC01 | ON227090 | Yes | Yes |
| DduNC12-001 | Yes | Yes | Yes | Noumea | STRANDING | 09/01/2012 | -22,240913 | 166,229818 | 260 | F | F | DduNC01 | ON227090 | Yes | Yes |
| DduNC12-002 | Yes | Yes | Yes | Voh | HUNTING | ND | -21,040447 | 164,697783 | NA | NA | F | DduNC01 | ON227090 | Yes | Yes |
| DduNC12-155 | Yes | Yes | Yes | Noumea | STRANDING | 25/09/2012 | -22,202147 | 166,354219 | 256 | M | M | DduNC02 | ON227091 | Yes | Yes |
| DduNC12-156 | Yes | Yes | Yes | Noumea | STRANDING | 16/10/2012 | -22,292734 | 166,589427 | 253 | M | M | DduNC01 | ON227090 | Yes | Yes |
| DduNC12-158 | Yes | Yes | Yes | Poum | STRANDING | 01/02/2012 | -20,081568 | 163,997705 | ≈200 | F | F | DduNC01 | ON227090 | Yes | Yes |
| DduNC12-159 | Yes | No | No | Noumea | STRANDING | 02/03/2012 | -22,31026 | 166,448061 | NA | NA | NA | DduNC01 | ON227090 | Yes | Yes |
| DduNC12-160 | Yes | Yes | Yes | La Foa | BIOPSY | 02/03/2012 | -21,829771 | 165,794068 | 270 | M | M | DduNC01 | ON227090 | Yes | Yes |
| DduNC12-161 | Yes | Yes | Yes | La Foa | BIOPSY | 03/03/2012 | -21,785926 | 165,681163 | 230 | F | F | DduNC01 | ON227090 | Yes | Yes |
| DduNC13-129 | Yes | Yes | Yes | Noumea | BIOPSY | 24/09/2013 | -22,289016 | 166,467 | 2,5 | M | M | DduNC03 | ON227092 | Yes | Yes |
| DduNC13-130 | Yes | Yes | Yes | Noumea | BIOPSY | 27/09/2013 | -22,3166 | 166,36666 | 280 | F | F | DduNC01 | ON227090 | Yes | Yes |
| DduNC13-131 | Yes | No | No | Noumea | BIOPSY | ND | -22,322183 | 166,3683 | NA | NA | M | DduNC01 | ON227090 | Yes | Yes |
| DduNC13-132 | Yes | Yes | Yes | Noumea | BIOPSY | 28/09/2013 | -22,327116 | 166,37951 | 260 | F | F | DduNC01 | ON227090 | Yes | Yes |
| DduNC13-134 | Yes | Yes | Yes | Bourail | BIOPSY | 01/10/2013 | -21,551646 | 165,19702 | 270 | F | F | DduNC01 | ON227090 | Yes | Yes |
| DduNC13-135 | Yes | Yes | Yes | Bourail | BIOPSY | 01/10/2013 | -21,528275 | 165,178389 | NA | F | F | DduNC01 | ON227090 | Yes | No |
| DduNC13-136 | Yes | Yes | Yes | Bourail | BIOPSY | 02/10/2013 | -21,5101 | 165,17909 | 290 | F | F | DduNC01 | ON227090 | Yes | Yes |
| DduNC13-137 | Yes | Yes | Yes | Bourail | BIOPSY | 02/10/2013 | -21,5089 | 165,1731 | 230 | F | F | DduNC01 | ON227090 | Yes | Yes |
| DduNC13-138 | Yes | Yes | Yes | Bourail | BIOPSY | 03/10/2013 | -21,52041 | 165,21535 | 220 | M | M | DduNC01 | ON227090 | Yes | Yes |
| DduNC13-139 | Yes | Yes | Yes | Bourail | BIOPSY | 03/10/2013 | -21,51386 | 165,20517 | 240 | M | M | DduNC01 | ON227090 | Yes | Yes |
| DduNC13-140 | Yes | Yes | Yes | Bourail | BIOPSY | 03/10/2013 | -21,52935 | 165,19276 | 270 | F | F | DduNC01 | ON227090 | Yes | Yes |
| DduNC13-141 | Yes | Yes | Yes | Bourail | BIOPSY | 04/10/2013 | -21,52844 | 165,21561 | 230 | M | M | DduNC01 | ON227090 | Yes | Yes |
| DduNC13-142 | Yes | Yes | Yes | Bourail | BIOPSY | 04/10/2013 | -21,54005 | 165,21538 | NA | M | M | DduNC01 | ON227090 | Yes | Yes |
| DduNC13-143 | Yes | Yes | Yes | Bourail | BIOPSY | 04/10/2013 | -21,53165 | 165,22276 | NA | M | M | DduNC01 | ON227090 | Yes | Yes |
| DduNC13-144 | Yes | Yes | Yes | Bourail | BIOPSY | 04/10/2013 | -21,5262 | 165,20306 | NA | F | F | DduNC01 | ON227090 | Yes | Yes |
| DduNC13-163 | Yes | Yes | Yes | Poum | HUNTING | ND | -20,226754 | 164,029328 | NA | NA | NA | DduNC01 | ON227090 | Yes | No |
| DduNC13-164 | Yes | Yes | Yes | Poum | HUNTING | ND | -20,233177 | 164,048577 | NA | NA | NA | DduNC01 | ON227090 | Yes | No |
| DduNC14-001 | Yes | No | No | Noumea | STRANDING | 10/02/2014 | -22,21525 | 166,356246 | 255 | M | M | DduNC01 | ON227090 | Yes | Yes |
| DduNC14-077 | No | No | No | Bourail | STRANDING | 07/02/2014 | -21,369426 | 165,103043 | ≈110 | NA | M | NA | NA | No | No |
| DduNC14-078 | Yes | Yes | Yes | Koumac | STRANDING | 24/11/2014 | -20,625434 | 164,320815 | ≈170 | M | M | DduNC01 | ON227090 | Yes | No |
| DduNC14-079 | Yes | Yes | Yes | La Foa | STRANDING | 23/07/2014 | -21,813811 | 165,766987 | 230 | F | F | DduNC01 | ON227090 | Yes | No |
| DduNC15-171 | Yes | Yes | Yes | Boulouparis | STRANDING | 02/07/2015 | -21,924873 | 165,952188 | ≈240 | F | F | DduNC01 | ON227090 | Yes | No |
| DduNC15-172 | Yes | No | No | La Foa | STRANDING | 09/12/2015 | -21,862007 | 165,815894 | 120 | NA | NA | DduNC01 | ON227090 | Yes | No |
| DduNC18-001 | Yes | No | No | Boulouparis | STRANDING | 22/01/2018 | -21,947536 | 166,126435 | ≈250 | NA | NA | DduNC01 | ON227090 | Yes | No |
| DduNC19-001 | Yes | No | No | La Foa | STRANDING | 09/01/2019 | -21,817839 | 165,767426 | 310 | M | M | DduNC01 | ON227090 | Yes | No |
| DduNC19-002 | Yes | Yes | Yes | Boulouparis | STRANDING | 19/06/2019 | -22,061142 | 166,0273 | 130 | M | M | DduNC01 | ON227090 | Yes | No |
| DduNC19-003 | Yes | Yes | Yes | La Foa | STRANDING | 01/08/2019 | -21,859368 | 165,864871 | 298 | F | F | DduNC01 | ON227090 | Yes | No |
| DduNC19-200 | Yes | Yes | Yes | Voh | BIOPSY | 04/10/2019 | -20,99966 | 164,65275 | 236 | F | F | DduNC01 | ON227090 | Yes | No |
| DduNC19-201 | Yes | Yes | Yes | Voh | BIOPSY | 10/10/2019 | -21,14301 | 164,72916 | 223 | F | F | DduNC01 | ON227090 | Yes | No |
| DduNC19-202 | Yes | Yes | Yes | Voh | BIOPSY | 10/10/2019 | -21,16979 | 164,74718 | 295 | F | F | DduNC01 | ON227090 | Yes | No |
| DduNC19-203 | Yes | Yes | Yes | Voh | BIOPSY | 11/10/2019 | -21,15866 | 164,74955 | 240 | F | F | DduNC01 | ON227090 | Yes | No |
| DduNC19-204 | Yes | Yes | Yes | Voh | BIOPSY | 11/10/2019 | -21,160833 | 164,74356 | 180 | F | F | DduNC01 | ON227090 | Yes | No |
| DduNC20-001 | Yes | Yes | Yes | Koumac | STRANDING | 03/01/2020 | -20,573902 | 164,288333 | ≈289 | NA | F | DduNC01 | ON227090 | Yes | No |
| DduNC20-002 | Yes | No | No | Noumea | STRANDING | 12/02/2020 | -22,16957 | 166,305971 | ≈200 | M | M | DduNC01 | ON227090 | Yes | No |
| DduNC20-013 | Yes | Yes | Yes | La Foa | STRANDING | 22/07/2020 | -21,851382 | 165,825695 | 265 | F | F | DduNC01 | ON227090 | No | No |
| DduNC20-014 | Yes | No | No | Boulouparis | STRANDING | 10/08/2020 | -21,982448 | 166,111929 | NA | M | M | DduNC01 | ON227090 | No | No |
| DduNC21-001 | Yes | Yes | Yes | Prony | STRANDING | ND |  |  | 105 | F | F | DduNC01 | ON227090 | No | No |
| DduNC21-002 | Yes | Yes | Yes | Bourail | STRANDING | 29/05/2021 | -21,613707 | 165,46114 | 270 | F | F | DduNC01 | ON227090 | No | No |
| DduNC21-003 | Yes | Yes | Yes | Bourail | STRANDING | 30/07/2021 | -21,604201 | 165,44326 | 263 | F | F | DduNC01 | ON227090 | No | No |
| DduNC21-004 | Yes | Yes | Yes | Voh | STRANDING | 08/10/2021 | -21,101557 | 164,827076 | 270 | M | M | DduNC01 | ON227090 | No | No |
| DduNC21-005 | Yes | Yes | No | Boulouparis | STRANDING | 25/10/2021 | -21,913397 | 166,078407 | 225 | M | M | DduNC01 | ON227090 | No | No |
| DduNC21-006 | Yes | No | No | Noumea | STRANDING | 29/10/2021 |  |  | nd | NA | NA | DduNC01 | ON227090 | No | No |
| DduNC22-002 | Yes | No | No | Boulouparis | STRANDING | 15/02/2022 | -21,937529 | 166,023878 | 301 | F | F | DduNC01 | ON227090 | No | No |
| DduNC23-002 | Yes | No | No | Poum | STRANDING | 15/02/2023 |  |  | 240 | NA | M | DduNC01 | ON227090 | No | No |
| DduNC23-001 | Yes | Yes | Yes | La Foa | STRANDING | 31/01/2023 |  |  | 136 | M | M | DduNC01 | ON227090 | No | No |
| DduNC23-014 | Yes | No | No | Noumea | STRANDING | 01/09/2023 |  |  | 244 | F | F | DduNC01 | ON227090 | No | No |

**
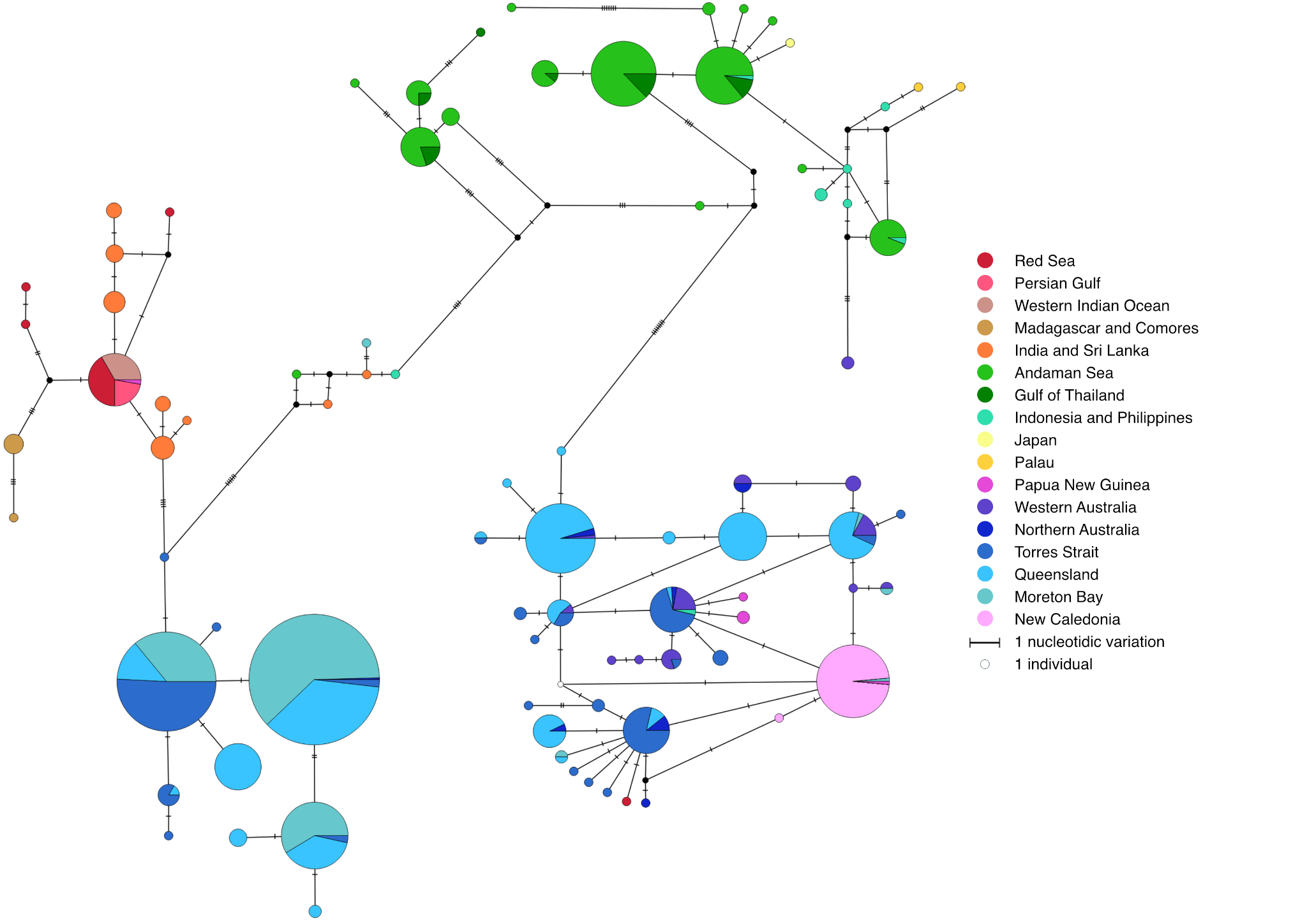
**

**Figure S1**: Median-joining haplotype network obtained by aligning 1,010 mitochondrial control region sequences from individuals across the global range of dugongs. The individuals included were from Western Australia (N = 28, Blair et al. 2014), Northern Australia (N = 11, Blair et al. 2014; Plön et al. 2019), the Torres Strait (N = 139, Blair et al. 2014; McGowan et al. 2023), Queensland (N = 293, Blair et al. 2014; Seddon et al. 2014; Plön et al. 2019; McGowan et al. 2023), Moreton Bay (N = 222, Blair et al. 2014; Seddon et al. 2014; Plön et al. 2019; McGowan et al. 2023), India and Sri Lanka (N = 26, Plön et al. 2019; Srinivas et al. 2021), Madagascar and the Comoros (N = 6, Plön et al. 2019), the Western Indian Ocean (N = 39, Plön et al. 2019), Southeast Asia (N = 172, Bushell et al. 2013; Blair et al. 2014; Plön et al. 2019; Poommouang et al. 2021), the Northwest Pacific (N = 3, Blair et al. 2014), Papua New Guinea (N = 5, Plön et al. 2019), and New Caledonia (N = 65, Garrigue et al. 2022; present study).


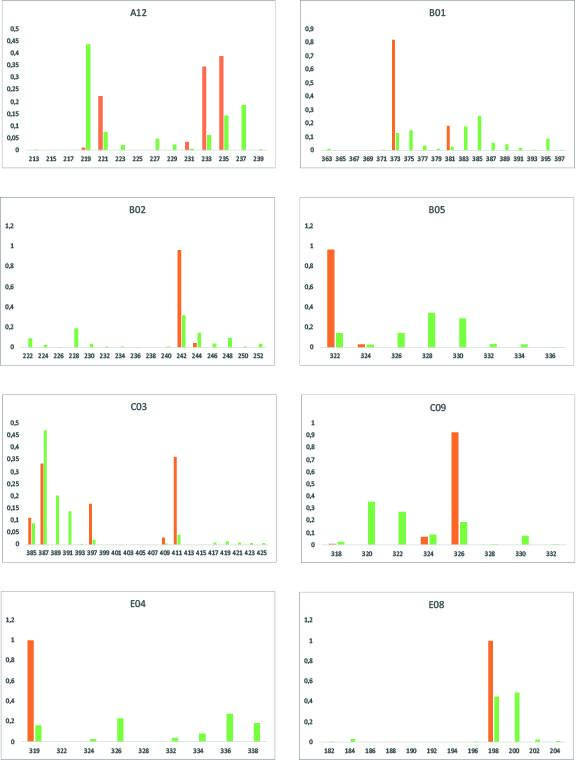


**Figure S2:** Allele frequency distributions at 13 microsatellite loci obtained from two datasets: New Caledonian dugongs (in orange) and Australian dugongs (in green) from a subset of McGowan et al. (2023).


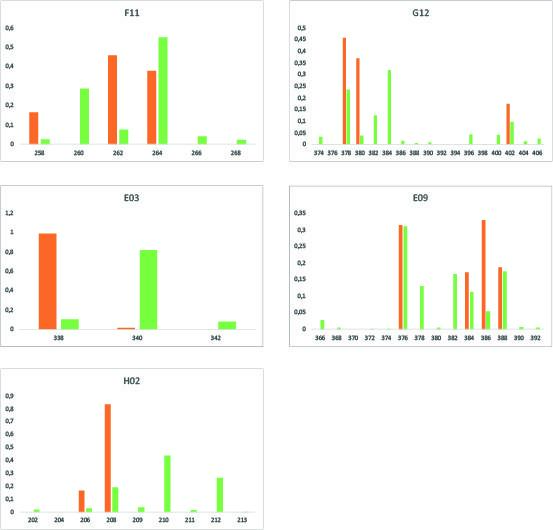


**Figure S2 (continuing)**

**
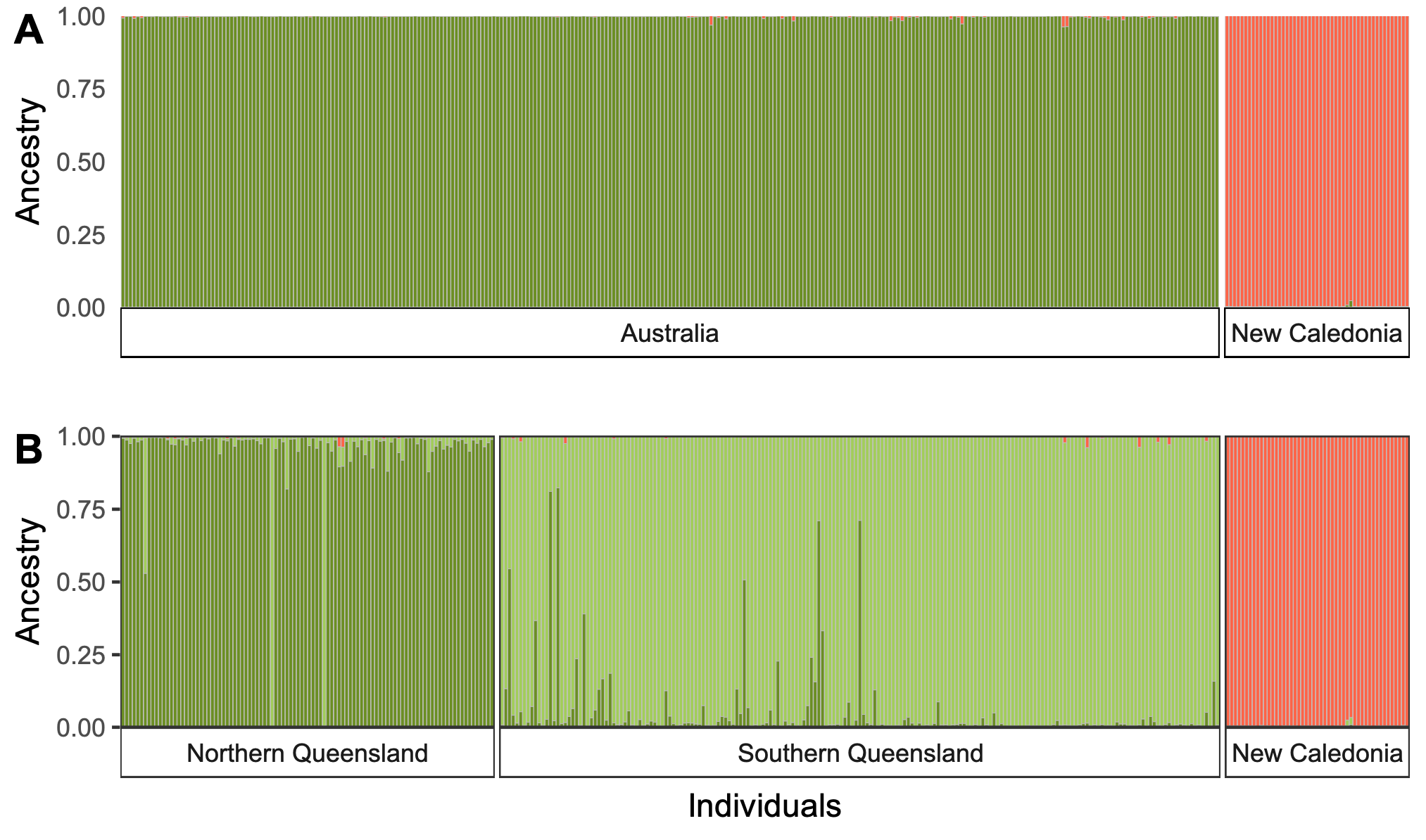
**

**Figure S3**: Genetic population structure of dugong in the Coral Sea: structure plot based on the analysis of 342 samples (N = 49 in New Caledonia; N = 293 in Australia) based on 13 microsatellite loci.


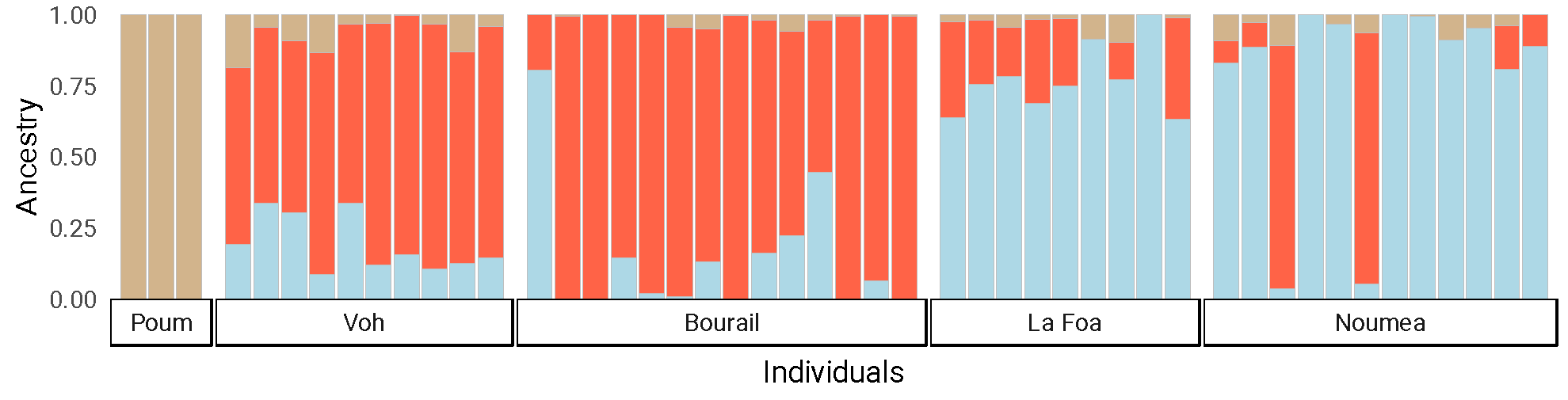


**Figure S4**: Individual admixture coefficients of New Caledonian dugongs based on 2,999 SNPs from the global population to the different clusters for a number of ancestral populations K =3 best fitting the data according to the *snmf* function.


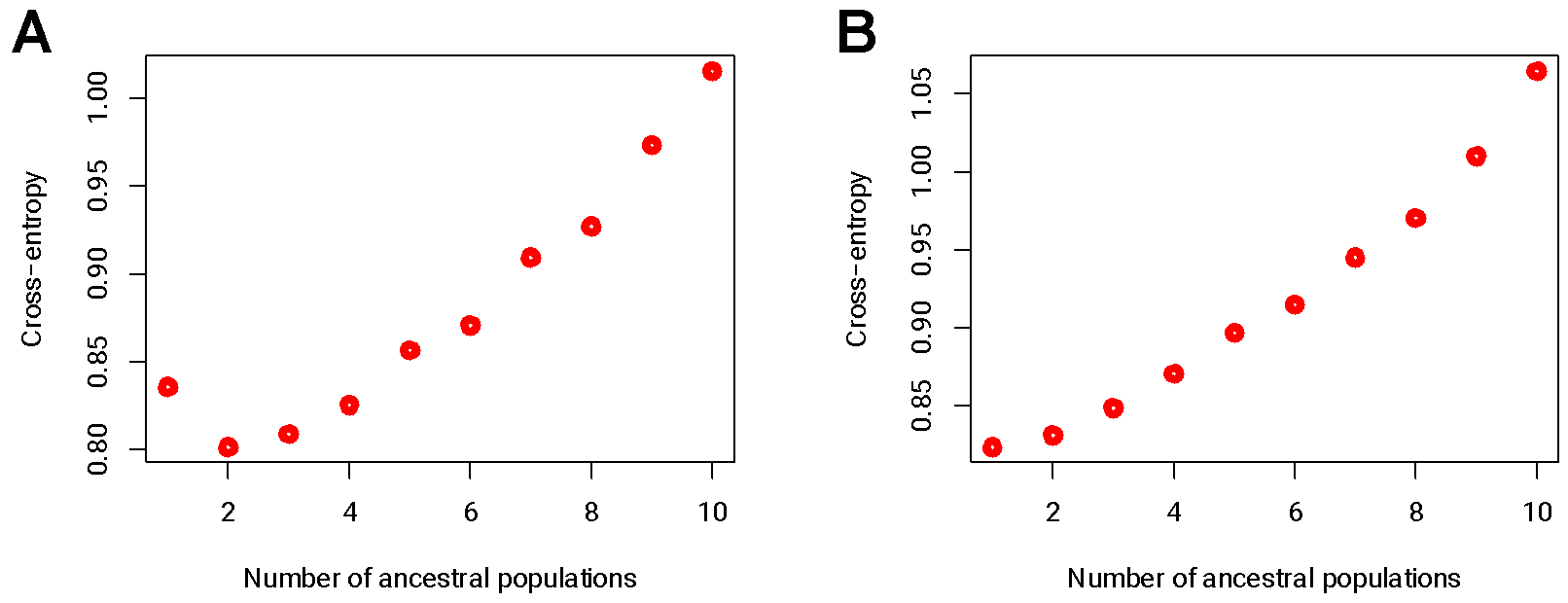


**Figure S5**: Entropy values of snmf analyses A) of the overall population and B) when excluding 2 related individuals from Poum.


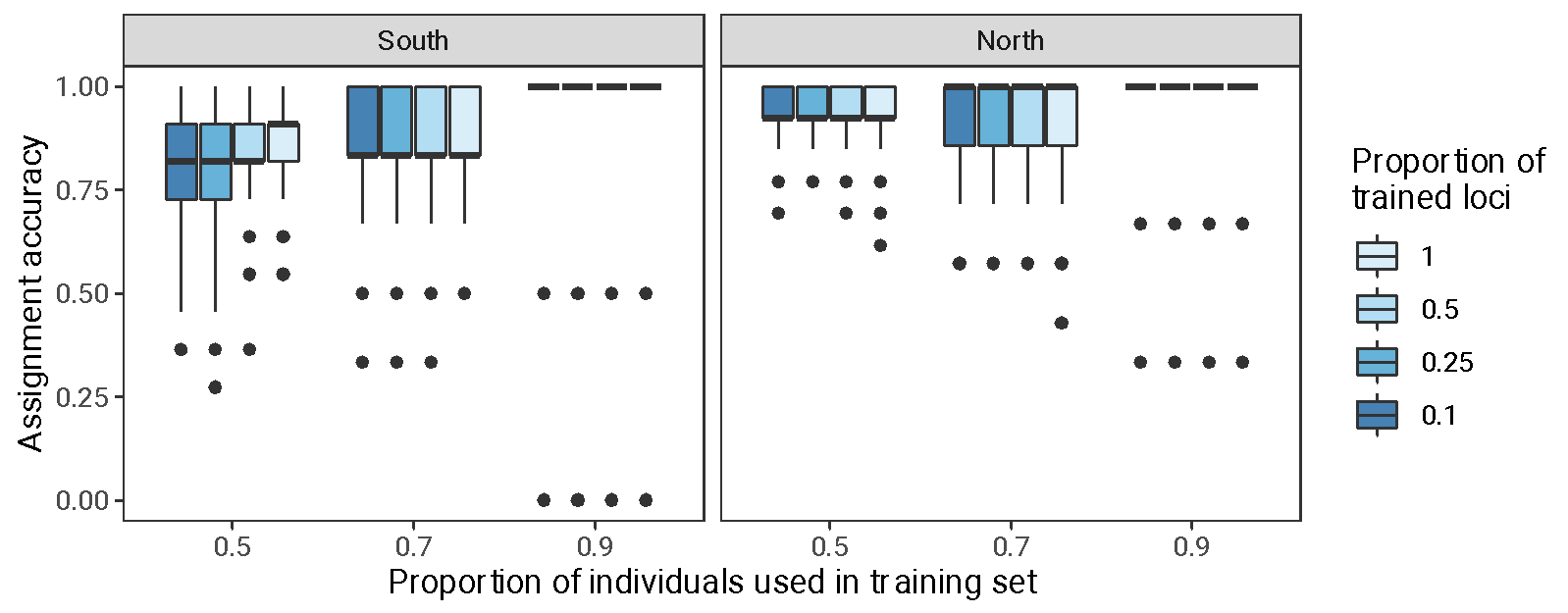


**Figure S6**: Accuracy of the assignment computed by the *assignPOP* R package for southern and northern sub-populations. The boxes show the variance of accuracy values for each interaction, the black points represent outliers values in accuracy.

**Cited references**

Blair, D., McMahon, A., McDonald, B., Tikel, D., Waycott, M., & Marsh, H. (2014). Pleistocene sea level fluctuations and the phylogeography of the dugong in Australian waters. *Marine Mammal Science*, *30*(1), 104‑121.<https://doi.org/10.1111/mms.12022>

Bushell, J. B. (2013). The genetic diversity and population structure of the dugongs (Dugong dugon) of Thailand. Master's Theses. 4379. <https://doi.org/10.31979/etd.9mna-xz4c>

Garrigue, C., Bonneville, C. D., Cleguer, C., & Oremus, M. (2022). Extremely Low mtDNA Diversity and High Genetic Differentiation Reveal the Precarious Genetic Status of Dugongs in New Caledonia, South Pacific. *Journal of Heredity*, *113*(5), 516‑524.<https://doi.org/10.1093/jhered/esac029>

Plön, S., Thakur, V., Parr, L., & Lavery, S. D. (2019). Phylogeography of the dugong (*Dugong dugon*) based on historical samples identifies vulnerable Indian Ocean populations. *PLOS ONE*, *14*(9), e0219350.<https://doi.org/10.1371/journal.pone.0219350>

Poommouang, A., Kriangwanich, W., Buddhachat, K., Brown, J. L., Piboon, P., Chomdej, S., Kampuansai, J., Mekchay, S., Kaewmong, P., Kittiwattanawong, K., & Nganvongpanit, K. (2021). Genetic diversity in a unique population of dugong (*Dugong dugon*) along the sea coasts of Thailand. *Scientific Reports*, *11*(1), 11624.<https://doi.org/10.1038/s41598-021-90947-4>

Seddon, J. M., Ovenden, J. R., Sneath, H. L., Broderick, D., Dudgeon, C. L., & Lanyon, J. M. (2014). Fine scale population structure of dugongs (*Dugong dugon*) implies low gene flow along the southern Queensland coastline. *Conservation Genetics*, *15*(6), 1381‑1392.<https://doi.org/10.1007/s10592-014-0624-x>

Srinivas Y, Pande A, Gole S, et al. Mitochondrial phylogeography reveals high haplotype diversity and unique genetic lineage in Indian dugongs (*Dugong dugon*). *Aquatic Conserv: Mar Freshw Ecosyst*. 2021; 31: 818–829.<https://doi.org/10.1002/aqc.3490>
